# SARS-CoV-2 virus NSP14 Impairs NRF2/HMOX1 activation by targeting Sirtuin 1

**DOI:** 10.1038/s41423-022-00887-w

**Published:** 2022-06-23

**Authors:** Shilei Zhang, Jingfeng Wang, Lulan Wang, Saba Aliyari, Genhong Cheng

**Affiliations:** Department of Microbiology, Immunology, and Molecular Genetics, University of California, Los Angeles, Los Angeles, 90095 CA USA

**Keywords:** SARS-CoV-2, NRF2, HMOX1, NSP14, SIRT1, Oxidative stress, Infection, Immune evasion

## Abstract

Most deaths from the COVID-19 pandemic are due to acute respiratory distress syndrome (ARDS)-related respiratory failure. Cytokine storms and oxidative stress are the major players in ARDS development during respiratory virus infections. However, it is still unknown how oxidative stress is regulated by viral and host factors in response to SARS-CoV-2 infection. Here, we found that activation of NRF2/HMOX1 significantly suppressed SARS-CoV-2 replication in multiple cell types by producing the metabolite biliverdin, whereas SARS-CoV-2 impaired the NRF2/HMOX1 axis through the action of the nonstructural viral protein NSP14. Mechanistically, NSP14 interacts with the catalytic domain of the NAD-dependent deacetylase Sirtuin 1 (SIRT1) and inhibits its ability to activate the NRF2/HMOX1 pathway. Furthermore, both genetic and pharmaceutical evidence corroborated the novel antiviral activity of SIRT1 against SARS-CoV-2. Therefore, our findings reveal a novel mechanism by which SARS-CoV-2 dysregulates the host antioxidant defense system and emphasize the vital role played by the SIRT1/NRF2 axis in host defense against SARS-CoV-2.

## Introduction

COVID-19 is an emerging worldwide infectious disease caused by a novel coronavirus, severe acute respiratory syndrome coronavirus 2 (SARS-CoV-2), which has led to a profound threat to global health [1]. Most of the fatalities caused by SARS-CoV-2 infection are due to acute respiratory distress syndrome (ARDS)-related respiratory failure, which is characterized by clinical symptoms such as cytokine storms, oxidative stress, shortness of breath, thrombosis, sepsis, and lung damage [2, 3]. Oxidative stress is a result of an imbalance between oxidant production and antioxidant mechanisms, and it causes metabolic and physiological alterations and various diseases in organisms [4]. Since the first evidence showing that RSV induces oxidative stress [5], numerous studies have verified the development of oxidative stress in various viral infections, including DNA virus (human papilloma virus and hepatitis B virus) and RNA virus (Japanese encephalitis virus, dengue virus, and influenza virus) infections [6, 7]. Some studies have also reported an association between oxidative stress and the severity of COVID-19 patients, indicating that oxidative stress amplifies and prolongs cytokine storm, coagulopathy, and cellular hypoxia [8, 9]. However, how SARS-CoV-2 infection causes oxidative stress is still unclear.

In response to aberrant oxidative stress, cells have different systems to defend against oxidative stress-derived damage and inflammation. In recent decades, nuclear factor erythroid 2–related factor 2 (Nrf2) has been identified as a master regulator of cellular resistance to oxidants, which is tightly regulated by Kelch-like ECH-associated protein-1 (Keap1) [10]. Numerous studies have been reported either the activation or suppression of Nrf2 in cells infected with different viruses [6]. Antonio et al. proposed that pharmacological activation of NRF2 can be deployed against SARS-CoV-2 to provide robust cytoprotection by restoring redox and protein homeostasis, promoting the resolution of inflammation and facilitating repair [11]. David Olagnier et al. revealed that the NRF2 agonists 4-octyl-itaconate (4-OI) and dimethyl fumarate (DMF) induce a cellular antiviral program that potently inhibits the replication of SARS-CoV-2 through a type-I interferon-dependent mechanism [12]. However, to date, the mechanisms by which NRF2-related signals defend against SARS-CoV-2 infection, as well as how SARS-CoV-2 engages in the antioxidant response, are still not fully understood.

SARS-CoV-2 has a single-stranded, positive-sense RNA genome of approximately 30 kb that encodes structural proteins (Spike, M, N, and E) and multiple nonstructural proteins (NSP1-NSP16) [13]. Among these proteins, the NSP14 protein is a bifunctional enzyme that harbors an N-terminal 3′ to 5′ exoribonuclease (ExoN) domain and a C-terminal S-adenosylmethionine (SAM)-dependent N7-methyltransferase (MTase) domain [14–16]. ExoN activity is crucial for the coronavirus replication proofreading activity that improves its genome stability and fidelity replication [17], while the N7-MTase activity has been postulated to be a key factor for 5’ cap methylation of the viral RNA genome, which enables viral evasion of host innate immunity [18–20]. The exonuclease activity of NSP14 is stimulated by the cofactor nsp10, whereas the N7-MTase activity of NSP14 does not depend on the cofactor NSP10 [21–23]. The ExoN and N7-MTase enzymatic activities of NSP14 are critical for inducing RNA modifications that are essential for viral RNA stability and translation as well as for preventing the activation of the host immune response, making it an attractive target for the development of new COVID-19 antiviral therapeutics [24–26].

In this study, we demonstrated that SARS-CoV-2 infection decreases antioxidant gene expression and that pharmacological activation of NRF2 signaling triggers a robust anti-SARS-CoV-2 effect. The metabolite biliverdin produced by HMOX1 can potently block SARS-CoV-2 replication in several tested cell lines. Furthermore, we performed an unbiased screening to identify the viral protein NSP14, which inhibits the expression of NRF2-mediated antioxidant genes by interacting with SIRT1. Correspondingly, SIRT1 boosts antioxidant response element (ARE)-dependent gene expression and exhibits a powerful antiviral effect against SARS-CoV-2. Collectively, these findings reveal a novel inhibitory function of SARS-CoV-2 NSP14 on NRF2-mediated signaling and might provide clues for use in the development of new strategies against SARS-CoV-2 infection.

## Materials and methods

### Cell culture and virus

Huh7, Vero-E6, Calu-3 and Caco-2 cells were purchased from ATCC and cultured in DMEM (Thermo Fisher) supplemented with 10% fetal bovine serum (FBS, Omega) and 1% penicillin–streptomycin (P/S, Gibco) at 37 °C in a 5% CO_2_ humidified atmosphere. SARS-CoV-2 virus was kindly provided by the NIH, and fluorescently tagged SARS-CoV-2 was kindly provided by Prof. Pei-yong Shi at the University of Texas Medical Branch (UTMB). SARS-CoV-2 virus was propagated in Vero-E6 cells and titrated on the basis of the TCID50. Cells were infected with SARS-CoV-2 at a multiplicity of infection (MOI) of 0.01 in Opti-MEM for 1 h and then cultured with fresh medium supplemented with 2% FBS. All experiments with the SARS-CoV-2 virus were performed in a BSL-3 laboratory at the UCLA.

### Antibodies and plasmids

The antibodies used in this study were as follows: Spike antibody from GeneTex (#GTX632604); antibodies against HMOX1 (#5853), GAPDH (#2118), GFP-Tag (#2555), HA-Tag (#3724) from Cell Signaling; antibody against HSP90 (sc-515081) from Santa Cruz; anti-Flag-tag antibody (#F1804) and anti-FLAG® M2 magnetic beads (#M8823) from Sigma; anti-tubulin antibody from Proteintech (#66031). Pierce™ anti-HA agarose from Thermo Fisher (#26181). Plasmids expressing the SARS-CoV-2 proteins were kindly provided by Prof. Peihui Wang (Shandong University, China). Full-length and truncated SIRT1 sequences were amplified from human cDNA and subcloned into a pCDNA-3xHA vector. Full-length NRF2 was amplified from pCDNA3-Myc3-Nrf2 (gift from Yue Xiong; Addgene plasmid #21555) and subcloned into pCDNA-3xHA. An ARE reporter kit (NRF2 antioxidant pathway) was purchased from BPS Bioscience (#60514). For transient expression, HEK293T cells were transfected with the indicated plasmids using the PEI method. After 24 h, cell lysates were prepared for further analysis. Briefly, HEK293T cells were seeded in 12-well plates for incubation at 37 °C and 5% CO_2_ for 20 h. The plasmids were added to 100 μL of Opti-MEM medium and briefly mixed. Then, the exact amount of 1 mg/mL PEI (ratio of DNA/PEI: 1 of μg DNA/3 μL of PEI) was added to 100 μL of solution with diluted plasmid, briefly mixed and maintained at room temperature for 20 min. Then, 100 μL of the PEI/DNA complex was added dropwise to wells, and the cells were incubated at 37 °C for 24 h.

### siRNA-mediated gene knockdown

The pooled siRNAs against human SIRT1 (sc-40986) and PGC-1α (sc-38884) were purchased from Santa Cruz Biotechnology. For siRNA transfection. siRNA against target genes were introduced into Huh7 cells using Lipofectamine RNAiMAX reagent (Thermo Fisher, USA) according to the manufacturer’s instructions and incubated for 48 h. Then the cells were infected with SARS-CoV-2 at an MOI of 0.01 for an additional 24 h or 48 h. Briefly, 1 μL of a siRNA duplex (10 μM) and 2 μL of RNAiMAX reagent were separately diluted into 50 μL of Opti-MEM medium and incubated at room temperature for 5 min, and then, 50 μL of siRNA duplex/Opti-MEM was added to 50 μL of RNAiMAX/Opti-MEM solution, briefly mixed and maintained at room temperature for an additional 20 min. The culture medium was replaced with 400 μL of Opti-MEM, and then, 100 μL of siRNA/RNAiMAX complexes were added dropwise to the plates and then incubated at 37 °C. Eight hours post-incubation, the siRNA/RNAiMAX was removed, and fresh medium with 2% FBS was added and then incubated at 37 °C for an additional 48 h.

### Inhibitors and compounds

The following inhibitors and compounds were used: hemin chloride (Santa Cruz Biotech, sc-202646); protoporphyrin IX cobalt chloride (Sigma, C1900); sulforaphane (Selleck, S5771); bardoxolone (MedChemExpress, HY-14909); tert-butylhydroquinone (Sigma, 112941); iron(II) chloride (Sigma, 372870); hemoglobin from bovine blood (Sigma, H2500); biliverdin hydrochloride (Sigma, 30891); SRT1720 hydrochloride (MedChemExpress, HY-15145); and protease inhibitor cocktail (Sigma, 11836153001).

### Immunofluorescence microscopy

Cells were washed with PBS and fixed with 4% paraformaldehyde in PBS for 15 min at room temperature. The cells were washed with PBS 3 times and then stained with 0.1 μg/ml DAPI (Thermo Fisher, 62248) for 5 min. Fluorescence was observed with a Leica Fluorescence Microscope, and images were captured and processed using LAS X Multichannel acquisition software.

### Luciferase reporter assay

HEK293T cells were seeded in 24-well plates and then cotransfected with ARE luciferase reporter vector constitutively expressing Renilla luciferase vector (60 ng/μl) and 200 ng of plasmid encoding SIRT1 or NSP14. After incubation for 24 h, the cells were treated with NRF2 agonists for 12 h. The cells were harvested, and cell lysates were used to determine luciferase activity with a Dual-Luciferase reporter assay system (Promega). The data represent relative firefly luciferase activity normalized to Renilla luciferase activity.

### RNA extraction and real-time PCR

Total RNA was extracted from Huh7 cells by using TRIzol reagent (Invitrogen) and reverse transcribed to cDNA with iScript cDNA Synthesis Kits (Bio-Rad). cDNA was prepared for real-time PCR by using iTaq Universal SYBR Green Supermix (Bio-Rad). The primers used in real-time PCR were as follows: HMOX1-forward, 5′-AAGACTGCGTTCCTGCTCAAC-3′, and HMOX1-reverse, 5′-AAAGCCCTACAGCAACTGTCG-3′; and GAPDH-forward, 5′- GAGTCAACGGATTTGGTCGT-3′, and GAPDH-reverse, 5′-TTGATTTTGGAGGGATCTCG-3′. The assays was performed with samples in a total volume of 20 μl, containing 10 μl of 2x iTaq Universal SYBR Green Supermix, 1 μl of cDNA, 0.5 μl of each primer (10 mM), and 8 μl of nuclease-free H_2_O. The transcript level of HMOX1 was normalized on the basis of the GAPDH control.

### Viral RNA purification and quantitative real-time PCR analysis

Vero-E6 cells were infected with SARS-CoV-2 at an MOI of 0.01 for 24 h or 48 h. Two hundred microliters of cell culture medium was harvested for viral RNA extraction with a High Pure Viral RNA kit (Invitrogen). RNA was eluted with 30 µl of RNase-free water and used as the template for RT–PCR quantification. For qPCR analysis, the following specific primers targeting the SARS-CoV-2 N gene were used: forward, 5′-TAATCAGACAAGGAACTGATTA-3′, and reverse, 5′-CGAAGGTGTGACTTCCATG-3′. RT–qPCR was performed using One Step TB Green PrimeScript RT–PCR Kit II (Takara) and the following cycling conditions: 42 °C for 5 min, 95 °C for 10 sec, and 40 cycles of 95 °C for 5 sec, followed by 60 °C for 30 sec.

### Protein sample preparation and immunoblotting

Whole-cell lysates were harvested in lysis buffer (50 mM Tris-Cl, pH 8.0; 5 mM EDTA; 150 mM NaCl; and 0.5% NP-40) supplemented with protease inhibitor cocktail and PMSF. For the collection of cell lysates after viral infection, 2xLDS sample buffer was added to the cell samples after removal of the culture medium and then denatured at 95 °C for 10 min. Lysates were electrophoresed in 10% polyacrylamide gels, and the separated proteins were transferred to a PVDF membrane, which was then subjected to immunoblotting with the indicated antibodies.

### Coimmunoprecipitation (Co-IP)

Cells were lysed with IP buffer (50 mM Tris-Cl, pH 8.0; 5 mM EDTA; 150 mM NaCl; and 0.5% NP-40) plus protease inhibitor cocktail and 1 mM DTT, 1 mM NaF, 1 mM PMSF and 2 mM Na_3_VO_4_. Whole-cell lysates were obtained by centrifugation at 12000 rpm for 15 min at 4 °C and then incubated with anti-FLAG® M2 magnetic beads or Pierce™ anti-HA agarose for 3 h at 4 °C. The immune complexes were then washed with cold IP buffer four times and separated by 10% SDS–PAGE and subjected to immunoblot analysis with the indicated antibodies.

### Statistical analysis

Experimental data are presented as the means ± standard deviation (SD). All statistical analyses were performed by two-tailed Student’s *t* test. Differences were considered statistically significant when the P values were less than 0.05. *P < 0.05 and **P < 0.01 for all the analyses.

## Results

### NRF2/HMOX-1 agonists inhibit SARS-CoV-2 replication in vitro

**Fig. 1:**
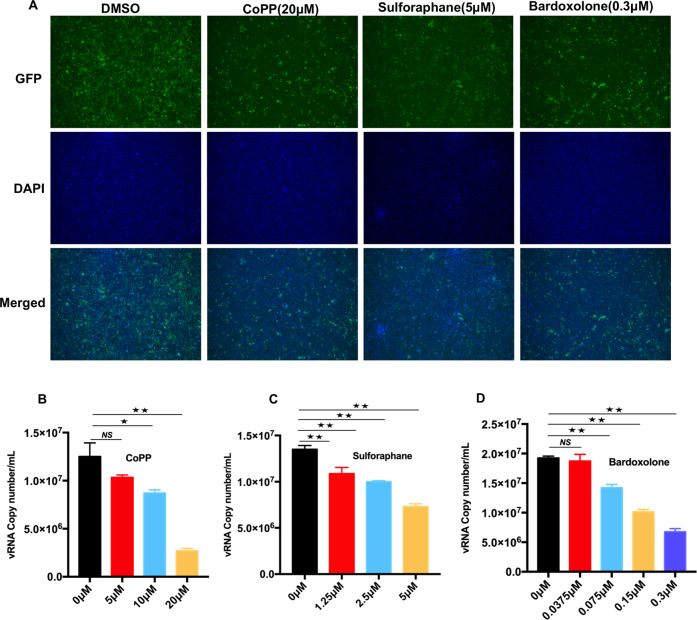
NRF2/HMOX1 agonists inhibit SARS-CoV-2 replication. **A** Fluorescence microscopy images of SARS-CoV-2 replication in NRF2/HMOX1 agonist-treated Vero-E6 cells. Vero-E6 cells were incubated with SARS-CoV-2 at an MOI of 0.01 for 1 h, and then treated with different NRF2/HMOX1 agonists, namely, cobalt protoporphyrin (CoPP), sulforaphane, or bardoxolone for 24 h. **B**, **D** The viral RNA copy number in the cell culture medium was determined by real-time PCR assay. The results are expressed as the mean ± SD (error bar) of 3 independent experiments; asterisks represent statistical significance based on two-tailed unpaired Student’s *t* test (*P  <  0.05, **P  <  0.01)

To determine the anti-SARS-CoV-2 activities of NRF2/HMOX-1 agonists, we first evaluated the viability of NRF2 agonist-treated Vero-E6 cells and HeLa-ACE2 cells (SI Appendix, Fig. S1) and confirmed that three NRF2/HMOX-1 agonists, cobalt protoporphyrin (CoPP), sulforaphane, and bardoxolone, increased the intracellular protein levels of NRF2 and HMOX1 (SI Appendix, Fig. S2A). Next, Vero-E6 cells were treated with increasing concentrations of NRF2/HMOX-1 agonists to evaluate their effects on SARS-CoV-2 replication. An immunofluorescence analysis showed that all NRF2/HMOX-1 agonists markedly inhibited SARS-CoV-2 replication (Fig. 1A). Moreover, to address whether NRF2 agonists affect the generation of SARS-CoV-2 viral particles, cell culture medium from infected Vero-E6 cells treated with agonists was harvested to quantify the viral RNA copies through real-time PCR. As presented in Fig. 1B–D, the NRF2 agonists displayed potent antiviral activity against SARS-CoV-2, as indicated by dose-dependent decreases in viral RNA copy numbers in the cell culture medium. Similar to that in Vero-E6 cells, viral replication was strongly influenced by NRF2 agonists in ACE2-HeLa cells (SI Appendix, Fig. S2B–E). To confirm the antiviral effects of NRF2/HMOX-1 agonists in cells derived from the lung, which is the primary target of SARS-CoV-2 infection, SARS-CoV-2 replication in Calu-3 human lung epithelial cells was examined by immunoblot quantification of the spike protein and viral RNA copies. As expected, CoPP treatment attenuated SARS-CoV-2 replication in a dose-dependent manner in the Calu3 cells (SI Appendix, Fig. S2F, G). The antiviral effect of CoPP was also observed in the Caco-2 colon carcinoma cells, in which Spike protein expression was reduced, and the number of progeny released into the cell culture medium was also reduced (SI Appendix, Fig. S2H–I). Taken together, these results demonstrated that NRF2/HMOX-1 agonists attenuated SARS-CoV-2 replication in multiple cellular systems.

### Biliverdin suppresses SARS-CoV-2 replication in vitro

**Fig. 2:**
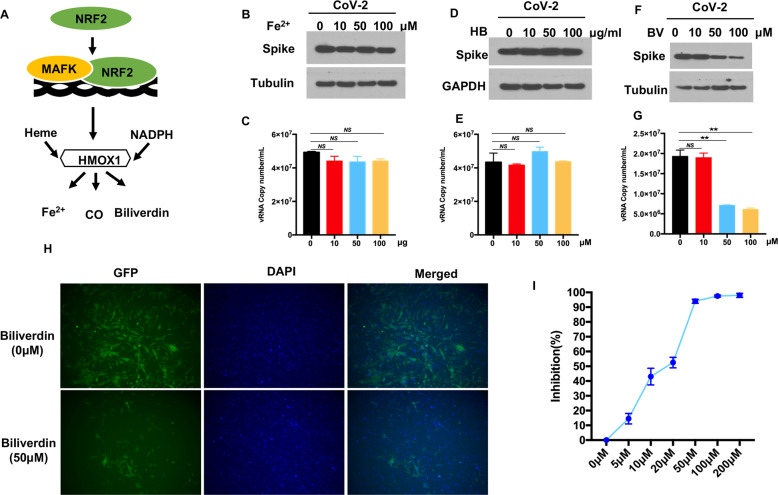
HMOX1 product biliverdin suppresses SARS-CoV-2 replication. **A** A schematic diagram showing the heme oxygenase pathway. Vero-E6 cells were infected with recombinant GFP-SARS-CoV-2 at an MOI of 0.01. After incubation with virus for 1 h, the cells were treated with the indicated doses of Fe^2+^ (**B)** hemoglobin (HB) **D** or biliverdin **(F)** for 24 h. **C**, **E**, **G** The amount of viral RNA in the cell culture medium was quantified by real-time PCR assay. **H** Fluorescence microscopy images of GFP-SARS-CoV-2 in biliverdin-treated ACE2-HeLa cells. Blue: DAPI (nuclear staining). **I** The dose response curve of biliverdin against SARS-CoV-2. HeLa-ACE2 cells were infected with recombinant GFP-SARS-CoV-2. After incubation with virus for 1 h, the cells were treated with biliverdin at different concentrations for an additional 24 h. The viral NP expression in the SARS-CoV-2-infected cells was quantified by real-time PCR and normalized to the expression of the 36B4 gene. The results are expressed as the mean ± SD (error bar) of 3 independent experiments; asterisks represent statistical significance based on two-tailed unpaired Student’s *t* test (*P  <  0.05, **P  <  0.01)

HMOX-1 is the rate-limiting enzyme that degrades heme to generate carbon monoxide, molecular iron and biliverdin, which can inhibit the replication of multiple viruses (Fig. 2A) [27]. To explore which of these metabolites is critical to the antiviral effect on SARS-CoV-2, Vero-E6 cells were challenged with SARS-CoV-2 in the presence of iron(II) chloride, the CO scavenger hemoglobin, or biliverdin. Interestingly, no significant changes were observed in the expression of spike and the number of NP copies, suggesting that CO and Fe^2+^ did not affect SARS-CoV-2 replication (Fig. 2B–E). In contrast, biliverdin cause a dose-dependent inhibition of SARS-CoV-2 replication, as evidenced by reduced spike protein expression and viral RNA copy numbers in the cell culture medium (Fig. 2F–G). Consistently, viral RNA copies and spike expression were much lower in biliverdin-treated CaCo-2, Huh7, and Vero-E6 cells than in the vehicle-treated control cells, indicating the potent antiviral activity of biliverdin (SI Appendix, Fig. S3A–F). Furthermore, we confirmed the antiviral effect of biliverdin on ACE2-HeLa cells. As shown in Fig. 2H, an immunofluorescence analysis of the GFP reporter virus showed that the number of fluorescent foci was lower in biliverdin-treated cells than in control cells. In addition, dose–response curves were determined on the basis of the quantity of viral RNA in the infected cells at 24 h post-infection. As demonstrated in Fig. 2I, biliverdin efficiently decreased the viral RNA levels in the infected ACE2-HeLa cells in a dose-dependent manner. Taken together, these data indicated that the metabolite biliverdin might be critical for the NRF2/HMOX-1 agonist-mediated inhibition of SARS-CoV-2 replication.

### SARS-CoV-2 NSP14 inhibits NRF2/HMOX-1 axis activation

**Fig. 3:**
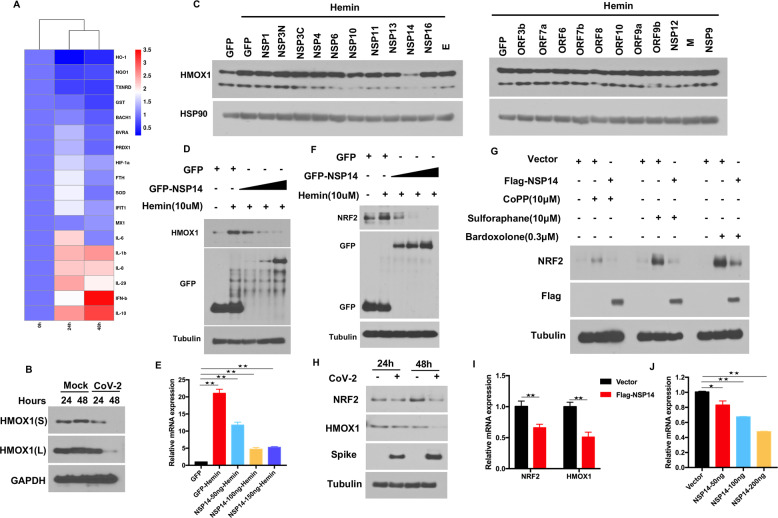
SARS-CoV-2 NSP14 suppresses NRF2/HMOX1 expression. **A** Heatmap showing comparisons of differential gene expression at 24 h and 48 h after SARS-CoV-2 infection of Huh7 cells. A bioinformatics analysis was performed using the OmicStudio tools at https://www.omicstudio.cn/tool. **B** Vero-E6 cells were challenged with SARS-CoV-2 for 24 h and 48 h. Endogenous HMOX1 was analyzed by immunoblotting. **C** Screening for viral proteins that inhibit HMOX1 expression. HEK293T cells were transfected with viral protein-expressing plasmids and then treated for 6 h with 10 μM hemin. Western blotting was performed to analyze the expression of endogenous HMOX1. **D** NSP14 inhibits HMOX1 expression in a dose-dependent manner. HEK293T cells were transfected with increasing amounts of plasmids expressing NSP14. Twenty-four hours post-transfection, the cells were treated with 10 μM hemin and subjected to an immunoblot analysis of endogenous HMOX1. **E** Real-time PCR analysis of HMOX1 mRNA. HEK293T cells were transfected with increasing amounts of plasmids expressing NSP14 for 24 h and then treated with 10 μM hemin for 6 h. Total RNA was extracted, and HMOX1 mRNA was determined by real-time PCR, and the level was normalized to that of GAPDH. **F**, **G** NSP14 inhibits NRF2 expression. HEK293T cells were transfected with the plasmid expressing NSP14. Twenty-four hours post-transfection, the cells were treated with the indicated agonists and subjected to an immunoblot analysis of endogenous NRF2. **H** Vero-E6 cells were challenged with SARS-CoV-2 for 24 h and 48 h. Endogenous NRF2 was analyzed by immunoblotting. **I, J)** HEK293T cells were transfected with increasing amounts of plasmids expressing NSP14 for 24 h. Total RNA was extracted, and NRF2 mRNA was determined by real-time PCR, and the level was normalized to that of GAPDH. Asterisks represent statistical significance based on two-tailed unpaired Student’s *t* test (*P  <  0.05, **P  <  0.01)

A previous study demonstrated that the NRF2 antioxidant gene expression pathway was suppressed in biopsy samples obtained from COVID-19 patients [12]. Here, we analyzed the antioxidation-related gene expression pattern upon SARS-CoV-2 infection. In contrast to that of enriched inflammation- and IFN-related genes, the expression of genes associated with the antioxidative response was widely suppressed by SARS-CoV-2, and among these genes, the expression of HMOX1 was profoundly downregulated at 24 h and 48 h post-infection (Fig. 3A). To confirm the suppression of HMOX1 expression by SARS-CoV-2, an immunoblot assay was performed to assess HMOX1 protein expression after SARS-CoV-2 challenge. The results showed that SARS-CoV-2 markedly decreased the protein expression of HMOX1, particularly at 48 h post-infection (Fig. 3B). Many viral proteins have been demonstrated to play important roles in regulating host signaling pathways [13, 28, 29]. For example, the Marburg virus VP24 protein interacts with Keap1 to activate the cytoprotective antioxidative response pathway [30]. To identify which proteins of SARS-CoV-2 regulate NRF2/HMOX-1 axis activation, we performed an unbiased screening to evaluate the effect of individual SARS-CoV-2 protein-expressing plasmids on HMOX1 expression. HEK293T cells were transiently transfected with plasmids expressing SARS-CoV-2 proteins and were subsequently stimulated with hemin, and endogenous HMOX1 expression was then determined. We found that hemin treatment induced endogenous HMOX1 expression, whereas overexpression of viral NSP14 but not that of other viral proteins profoundly inhibited HMOX1 expression (Fig. 3C). Furthermore, we transfected increasing amounts of NSP14-expressing plasmid into cells and found that NSP14 inhibited hemin-induced HMOX1 expression in a dose-dependent manner (Fig. 3D). To confirm the impact of NSP14 on HMOX1 expression, we examined HMOX1 expression induced by CoPP. Consistent with the abovementioned results, NSP14 also inhibited CoPP-induced HMOX1 expression, while NSP13 and NSP16 did not affect HMOX1 expression (SI Appendix, Fig. S4A).

The transactivation of HMOX1 and other antioxidant genes is regulated by binding of the transcription factor NRF2 to the ARE located at in the gene promoter regions [31]. We therefore postulated that NSP14 inhibits HMOX1 expression by affecting NRF2 expression. To test this hypothesis, we first measured the mRNA expression of HMOX1 in cells transfected with the NSP14 expression plasmid. A RT–qPCR analysis demonstrated that HMOX-1 mRNA expression induced by hemin (Fig. 3E) and CoPP (SI Appendix, Fig. S4B) was markedly downregulated by NSP14, indicating that NSP14 might target the transcription factor NRF2 to regulate downstream gene expression. We further analyzed NRF2 protein expression and found that NSP14 also suppressed hemin-induced NRF2 expression in a dose-dependent manner (Fig. 3F). In addition to that induced by hemin, the expression levels of various agonist-induced NRF2 proteins were all reduced by NSP14 (Fig. 3G). Moreover, SARS-CoV-2 infection led to decreased protein expression of endogenous NRF2, particularly at 48 h post-infection (Fig. 3H). Endogenous NRF2 mRNA expression was also evaluated through real-time PCR. Interestingly, the transcriptional levels of endogenous NRF2 and HMOX1 were both reduced upon overexpression of NSP14 (Fig. 3I–J). An ARE luciferase reporter system was designed to monitor the activity of the NRF2 antioxidant pathway in cultured cells. Since NRF2 agonists such as sulforaphane trigger the accumulation of NRF2 by modifying cysteine residues in the E3 ligase KEAP1, leading to a conformational change and disruption of the KEAP1-NRF2 interaction [32], HEK293T cells were transfected with the ARE luciferase reporter system along with a control vector or NSP14 and were then stimulated with the NRF2 agonist sulforaphane. As shown in Fig. S4C, overexpression of NSP14 significantly attenuated ARE-luciferase activity. Collectively, these results suggest that NSP14 is a novel viral protein that interferes with the NRF2-mediated antioxidant pathway.

### NSP14 interacts with SIRT1 and inhibits SIRT1-mediated NRF2 signaling

**Fig. 4:**
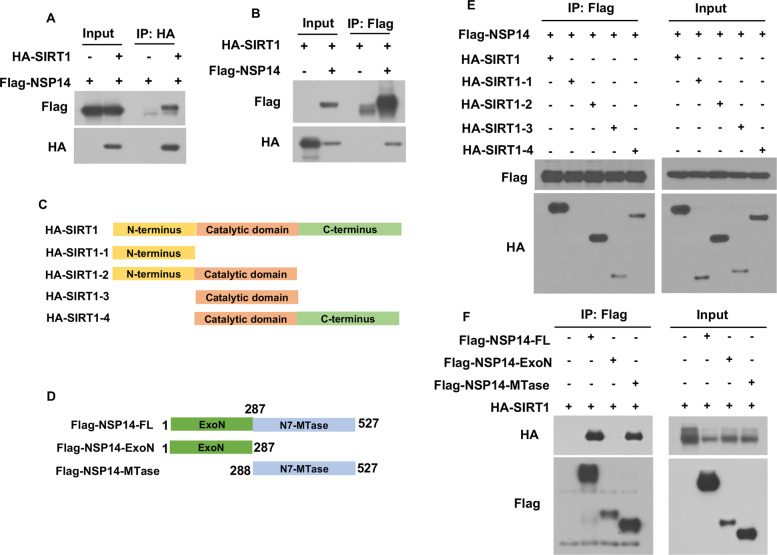
NSP14 interacts with SIRT1. **A**, **B** Detection of the interaction between NSP14 and SIRT1 by Co-IP assay. HEK293T cells were transfected with HA-SIRT1 and Flag-NSP14 for 24 h. The protein extracts were immunoprecipitated by anti-Flag magnetic beads or anti-HA agarose and then subjected to immunoblotting with the indicated antibodies. **C**, **D** Schematic representation of various truncations of HA-SIRT1 and Flag-NSP14 used for coimmunoprecipitation analyses. **E** A coimmunoprecipitation (Co-IP) assay was performed to examine the interaction between Flag-NSP14 and different truncations of HA-SIRT1. Co-IP was performed with anti-Flag magnetic beads, followed by WB with anti-HA antibody. **F** HA-SIRT1 was introduced into HEK293T cells with full-length NSP14 and different truncations. At 24 h post-transfection, protein samples were harvested for Co-IP assays using anti-Flag magnetic beads. Immunoblotting analysis was performed with the indicated antibodies

To investigate the mechanism by which NSP14 inhibits NRF2-mediated antioxidant pathway activation, we further analyzed an interaction network involving NSP14 and host proteins that was established on the basis of published mass spectrometry data [33]. Interestingly, we found that NSP14 bound the sirtuin family proteins SIRT1 and SIRT5 (SI Appendix, Fig. S5A). SIRT1 was predominantly located in the nucleus and cytosol, whereas SIRT5 was located in mitochondria [34]. Since NSP14 was detected in both the nucleus and cytosol [35], we postulated that NSP14 might interact with SIRT1 to regulate NRF2. To test this hypothesis, a co-IP assay was carried out after HEK293T cells were cotransfected with Flag-NSP14 and HA-SIRT1. Cell lysates were precipitated with anti-flag antibody beads or anti-HA antibody agarose. Immunoprecipitation with antibodies targeting either Flag or HA resulted in the pulldown of both proteins, indicating a physical interaction between NSP14 and SIRT1 (Fig. 4A, B). The data from the immunofluorescence assays showed that NSP14 colocalized with SIRT1, confirming the co-IP results (SI Appendix, Fig. S5B).

SIRT1 contains N-terminal, catalytic core, and C-terminal domains. To determine the domain that governs the interaction of SIRT1 with NSP14, we generated a series of SIRT1 truncation mutations and then carried out co-IP analysis using these mutant constructs (Fig. 4C). The N-terminal domain made no contribution to the association of SIRT1 with NSP14; however, other truncation mutants carrying the catalytic core showed the capacity to bind NSP14, suggesting that the catalytic core of SIRT1 is necessary for SIRT1 association with NSP14 (Fig. 4E). To determine the specific domain through which SIRT interacts with NSP14, full-length NSP14 and different truncation mutants (ExoN domain, MTase domain) were coexpressed with SIRT1 in HEK293 T cells, and then, a co-IP assay was performed (Fig. 4D). The results indicated that full-length NSP14 and the NSP14-MTase domain interacted with SIRT1 but that the ExoN domain of NSP14 did not interact with SIRT1 (Fig. 4F). Therefore, the interaction between NSP14 and SIRT1 was proven, and the binding domains were found to be the catalytic domain of SIRT1 and the MTase domain of NSP14.

**Fig. 5:**
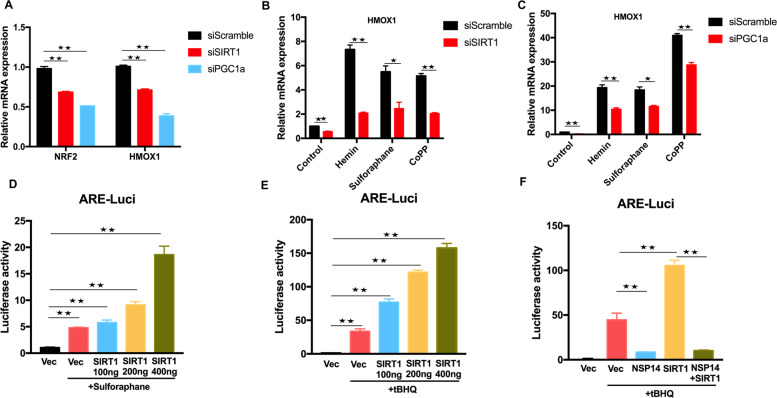
NSP14 inhibits SIRT1-mediated NRF2 signaling.**A** Real-time PCR analysis of NRF2 and HMOX1 mRNA expression. Huh7 cells were transfected with pooled siRNA against SIRT1 or PGC-1α for 48 h. Scramble siRNA was used as the control. Relative transcription levels of NRF2 and PGC-1α were quantified by real-time PCR and normalized to the level of GAPDH. **B, C** Real-time PCR analysis of HMOX1 mRNA expression mediated by different NRF2/HMOX1 agonists. Forty-eight hours post-transfection with siRNA against SIRT1 or PGC-1α, Huh7 cells were treated with hemin (10 μM), sulforaphane (10 μM), and CoPP (5 μM) for 6 h and then subjected to real-time PCR. **D, E** ARE-luciferase assays. HEK293T cells were transfected with Renilla luciferase control plasmid, firefly luciferase reporter plasmid ARE-Luc, and increasing amounts of SIRT1-expressing plasmid for 24 h and then treated with sulforaphane and tert-butylhydroquinone (tBHQ) for an additional 24 h. The protein samples were harvested for luciferase detection. **F** HEK293T cells were transfected with the Renilla luciferase control plasmid, firefly luciferase reporter plasmid ARE-Luc, HA-SIRT1, and Flag-NSP14 for 24 h following treatment with tBHQ for an additional 12 h. Then, cells were harvested for luciferase assay. Asterisks represent statistical significance based on two-tailed unpaired Student’s *t* test (*P  <  0.05, **P  <  0.01)

The above results showed that NSP14 can suppress the mRNA transcription of NRF2 and HMOX1 (Fig. 3). Therefore, we further determined the role played by SIRT1 in NRF2-related signaling. Silencing SIRT1 by siRNA markedly reduced the basal mRNA expression of NRF2 and HMOX1 as well as that of HMOX1 induced by different NRF2 agonists (Fig. 5A, B). In contrast, overexpression of SIRT1 enhanced ARE-luciferase activity induced by NRF2 agonists, sulforaphane, and tBHQ in a dose-dependent manner (Fig. 5D, E). Since NSP14 interacts with SIRT1, we found that NSP14 significantly inhibited the synergistic effect of NRF2 and SIRT1 on ARE-luciferase activity (Fig. 5F). SARS-CoV-2 NSP14 has been reported to inhibit translation to block IFN-I-dependent ISG induction through either ExoN or N7-MTase enzyme activity [19]. Structural studies of NSP14 have revealed that the exoribonuclease catalytic center is composed of five residues: D90, E92, E191, H268 and D273 [36–38]. We generated three catalytically inactive NSP14 mutants, D90A/E92A, H268A, and D90A/E92A/H268A (SI Appendix, Fig. S5C). ARE-luciferase assays showed that none of these mutations in the ExoN active sites abolished the inhibitory activity of NSP14 on tBHQ-induced ARE luciferase activity (SI Appendix, Fig. S5D).

### The SIRT1/PGC-1α/NRF2 pathway inhibits SARS-CoV-2 replication

**Fig. 6:**
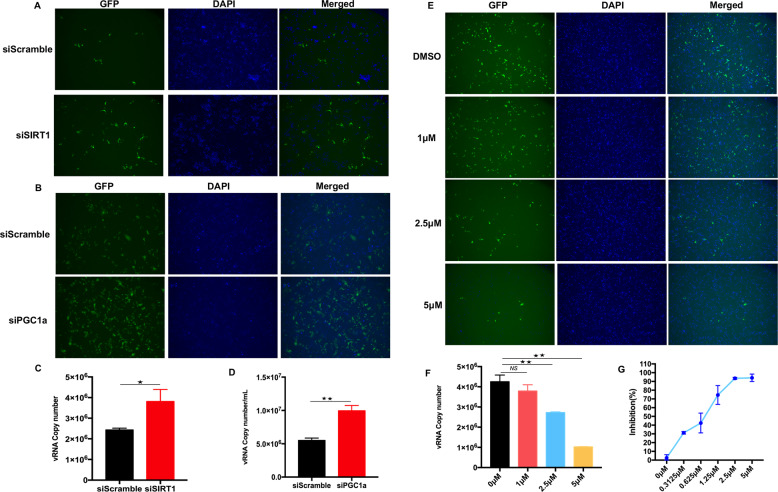
SIRT1/PGC-1α inhibits SARS-CoV-2 replication. **A**, **B** Knockdown of SIRT1/PGC-1α promotes SARS-CoV-2 replication. Huh7 cells were transfected with pooled siRNA against SIRT1 or PGC-1α for 48 h following challenge with infection with GFP-SARS-CoV-2 at an MOI of 0.01 for an additional 48 h. Microscopic imaging was performed to analyze SARS-CoV-2 replication. Blue: DAPI (nuclear staining). **C**, **D** The cell culture medium from (**A**, **B**) was also harvested for quantification of viral RNA copies by real-time PCR assay. **E** A SIRT1 agonist inhibits SARS-CoV-2 replication. Huh7 cells were incubated with GFP-SARS-CoV-2 at an MOI of 0.01 for 1 h and then treated with the SIRT1 agonist SRT1720 at the indicated doses for 48 h. Microscopy imaging was performed to analyze SARS-CoV-2 replication. Blue: DAPI (nuclear staining). **F** Real-time PCR assays were performed to quantify the copy number of viral RNA in the cell culture supernatants. **G** The dose response curves of SRT1720 against SARS-CoV-2. Huh7 cells were infected with GFP-SARS-CoV-2 for 1 h and then treated with the SIRT1 agonist SRT1720 at the indicated doses for 24 h. Cells were harvested for total RNA extraction. Viral NP expression was quantified by real-time PCR and then normalized to the expression of the 36B4 gene. Asterisks represent statistical significance based on two-tailed unpaired Student’s *t* test (*P  <  0.05, **P  <  0.01)

Since SIRT1 positively regulates NRF2-mediated signaling, the association between SIRT1 and SARS-CoV-2 replication was subsequently investigated. We performed siRNA-mediated knockdown experiments to determine the effects of SIRT1 on SARS-CoV-2 replication. An immunofluorescence microscopy image analysis showed that compared with the negative control siRNA, siRNA against SIRT1 promoted SARS-CoV-2 replication in Huh7 cells (Fig. 6A). Furthermore, the viral RNA copies in the cell culture medium of the SIRT1-knockdown cells was increased compared with those in the control cells (Fig. 6C). SRT1720 was developed as a potent activator of SIRT1, and it has been reported that SIRT1 activation by SRT1720 prolonged the lifespan and improved the metabolic status of various mouse models [39, 40]. Hence, we tested the anti-SARS-CoV-2 activity of SRT1720. After sufficient incubation, SRT1720 inhibited SARS-CoV-2 replication in both Huh7 cells (Fig. 6F) and ACE2-HeLa cells (SI Appendix, Fig. S6B) in a dose-dependent manner, as determined by the quantification analysis of viral RNA copies in the respective cell culture medium. The dose-dependent antiviral activity of SRT1720 was also evaluated through the quantification of viral NP expression in SARS-CoV-2-infected cells (Fig. 6G). In addition, the inhibitory activity of SRT1720 was confirmed with the visualization of GFP expression by immunofluorescence microscopy (Fig. 6E, SI Appendix, Fig. S6A).

Multiple targets of SIRT1 have been identified [41–45]. Among these targets, PGC-1α is a powerful regulator of NRF1 and NRF2 gene expression [46, 47], and SIRT1 requires PGC-1α to induce the expression of oxidative stress-protecting genes [48]. Consistent with the changes in NRF2 and HMOX1 expression in the SIRT1-knockdown cells, the mRNA transcription of NRF2 and HMOX1 was also downregulated when PGC-1α expression was silenced by siRNA (Fig. 5A and C). To explore the role played by PGC-1α in SARS-CoV-2 infection, we further examined the effect of PGC-1α knockdown on viral replication. As shown in Fig. 6B, the expression of GFP was increased in the PGC-1α-knockdown cells, as revealed by microscopy analysis. The viral RNA in the cell culture medium from SARS-CoV-2-infected WT and PGC-1α-knockdown cells 24 h post-infection was quantified through real-time PCR. The results showed that the number of viral RNA copies was significantly increased in the PGC-1α-knockdown cell culture medium (Fig. 6D). Collectively, these results indicated that the SIRT1/PGC-1α/NRF2/HMOX1 pathway is a host inhibitory pathway with activity against SARS-CoV-2.

## Discussion

COVID-19 is a pandemic disease with various syndromes ranging from fever, cough, headaches, sore throat, shortness of breath to more severe tissue damage and organ failure [49, 50]. As a severe acute respiratory disease caused by SARS-CoV-2 viral infection, many COVID-19 patients worldwide present with changes in blood oxygen levels and accumulated reactive oxygen species (ROS), which may lead to tissue damage and inflammatory responses [51]. The NRF2/HMOX1 axis, a major cytoprotective factor against ROS, is predominantly expressed in the respiratory tract, especially in lung tissue, and plays an important role in mediating anti-inflammatory activity and protecting tissue from damage caused by oxidative stress [52–54]. In addition to its well-known function in the oxidative stress response and heme metabolism, the NRF2/HMOX1 axis and its agonists exert inhibitory effects on a broad spectrum of viruses, as shown in our current study and that of others, which have expanded our knowledge. Furthermore, we provided evidence that biliverdin, a byproduct of heme metabolism produced by HMOX1, is critical for the antiviral function of NRF2/HMOX1 agonists against SARS-CoV-2. In this study, we also identified that SARS-CoV-2-encoded NSP14 interferes with the activation of the NRF2/HMOX1 axis by interacting with SIRT1, thus disrupting the NRF2/HMOX1-mediated antiviral response, which ultimately might lead to viral pathogenesis.

As an inducible defense system against oxidative stress, NRF2/HMOX1 basal expression is relatively low but can be markedly upregulated in response to various stimuli, such as hypoxia, cytokines, LPS, ROS, and infection [55–57]. Activation of NRF2 signaling has been observed in human cells infected with multiple viruses, including influenza, Marburg, Dengue, and Hepatitis B [6], while respiratory syncytial virus induces NRF2 degradation through an RNF4-dependent pathway [58]. These studies have suggested that NRF2 signaling can be regulated in response to viral infections. Our findings revealed that SARS-CoV-2 infection markedly decreases the activation of the NRF2/HMOX1 axis. Similar to our observations, the findings by David Olagnier et al. indicated that SARS-CoV-2 infection can suppress the expression of oxidative stress-related genes, including NRF2, HMOX1, and NADPH quinone oxidoreductase 1 (NqO1) [12]. We and others have demonstrated the antiviral activity of multiple NRF2 agonists, including CoPP, sulforaphane, bardoxolone, 4-octyl-itaconate (4-OI), dimethyl fumarate, and PB125 [12, 59, 60]. These studies have suggested that inhibiting NRF2 signaling might be involved in the pathogenesis of SARS-CoV-2 infection and that pharmaceutic activation of NRF2 signaling may be a therapeutic strategy for COVID-19.

Nrf2 is a master transcription factor that protects cells against oxidative stress by inducing the expression of a wide array of genes involved in immunity and inflammation, including those with antiviral action [61]. David Olagnier et al. demonstrated that the NRF2 agonist 4-OI induced a cellular antiviral program that potently inhibited the replication of SARS-CoV-2 through a type I interferon-independent mechanism [12]. Joe M. McCord et al. found that the NRF2-activating component PB125 downregulated ACE2 and TMPRSS2 mRNA expression in human liver-derived HepG2 cells and inhibited the transcriptional expression of inflammation-related genes [59]. In this study, we found that biliverdin, the byproduct of heme metabolism triggered by HMOX-1, can suppress SARS-CoV-2 replication in different cell lines, including Vero cells (type-I interferon defect cells). Considering that both 3CL-Pro and PLpro encoded by SARS-CoV-2 share high homology with other viral proteases that are known to be inhibited by biliverdin, biliverdin is expected to inhibit both SARS-CoV-2 3CLpro and PLpro expression [11, 62]. Annachiara et al. reported that SARS-CoV-2 spike bound biliverdin and bilirubin with nanomolar affinity, thus dampening the reactivity of the SARS-CoV-2 spike protein in immune sera and inhibiting the activity of a subset of neutralizing antibodies in an in vitro system [63]. Liu et al. found that serum bilirubin levels were correlated with COVID-19-induced liver injury and hemolysis [64]. However, our results showed potent antiviral activity of biliverdin against SARS-CoV-2 in different cell lines. Moreover, one Gilbert syndrome patient with hyperbilirubinemia exhibited rapid clinical recovery and endured only a short hospital stay compared to other COVID-19 patients, suggesting that hyperbilirubinemia may exert a protective effect against COVID-19-induced cardiometabolic disturbances [65]. Considering these controversial findings, further exploration of the biological function of biliverdin in COVID-19 is warranted.

Through its N-terminal 3′-5′ exoribonuclease (ExoN) and C-terminal N7-methyltransferase (N7-MTase) domains, NSP14 has been identified as a bifunctional protein that is not only involved in viral replication fidelity and viral RNA 5′ capping but also in the regulation of host innate immune responses [66]. Jack Chun-Chieh Hsu et al. showed that NSP14 was a virus-encoded translation inhibitory factor that abolished the type I interferon-dependent induction of interferon-stimulated genes (ISGs) [19]. Furthermore, NSP14 induced the lysosomal degradation of IFNAR1, thereby preventing STAT transcription factor activation [67]. In addition, NSP14 has also been reported to contribute to the viral activation of NF-κB signaling, which was identified by the nuclear translocation of the NF-κB p65 subunit and upregulation of IL-6 and IL-8 [68]. Mechanistically, NSP14 interacts with host inosine-5’-monophosphate dehydrogenase 2 (IMPDH2) protein, which is known to regulate NF-κB signaling [68]. Since the NRF2/HMOX1 axis has been established as a potent anti-inflammatory target counteracting the NF-κB-driven inflammatory response, our findings showing that NSP14 is a novel inhibitory factor in the antioxidative function of the NRF2/HMOX1 axis by interacting with SIRT1 may suggest a potential mechanism by which SARS-CoV-2 hyperactivates inflammatory NF-κB signaling.

SIRT1, a member of the NAD + -dependent sirtuin family (SIRT1-SIRT7), has been demonstrated to be a broad-spectrum and evolutionarily conserved host inhibitory factor with action against multiple viruses, including DNA viruses (HCMV, HSV-1, and adenovirus) and RNA viruses (influenza, enterovirus 71, and HTLV-1), through different mechanisms [69–71]. Our study revealed that SIRT1 is also a host inhibitory factor of SARS-CoV-2, as indicated by the increase in NRF2-dependent signaling. Moreover, SARS-CoV-2 NSP14 interacts with the deacetylase domain of SIRT1 and impairs the ability of SIRT1 to enhance NRF2 signaling. A similar working model has been reported for the study of human immunodeficiency virus type 1. Hye-Sook Kwon et al. found that the HIV Tat protein inhibited SIRT1 activity and induced T-cell hyperactivation through hyperacetylation of NF-κB [72]. In addition to SIRT1, other sirtuin family proteins have also been reported to regulate NRF2-dependent gene expression. SIRT2 deacetylates NRF2 on lysine 506 and 508, leading to a reduction in total and nuclear NRF2 levels [73]. SIRT6 is critical for activating the transcription of NRF2-responsive genes under oxidative stress through the mono-ADP-ribosylation of BAF170 [74]. Further research is needed to determine whether additional sirtuin family members also impede SARS-CoV-2 replication and whether NSP14 can antagonize sirtuin activity.

In conclusion, our study characterized the interplay between SARS-CoV-2 and the host SIRT1/NRF2/HMOX1 axis and provided mechanistic insight into the evasion of SARS-CoV-2 mediated by viral NSP14. These results not only advance our understanding of the pathogenesis of SARS-CoV-2 but also suggest that the SIRT1/NRF2/PGC-1α/HMOX1 axis can be used for the future development of a potential therapeutic target against SARS-CoV-2.

## Supplementary information

Supplemental Materials
