# Supplementary material for: SARS-CoV-2 virus NSP14 Impairs NRF2/HMOX1 activation by targeting Sirtuin 1
Source: Cell Mol Immunol. 2022 Jun 23;19(8):872–82. doi: 10.1038/s41423-022-00887-w (PMC9217730; doi:10.1038/s41423-022-00887-w)
Supplement: Supplementary file 1 — Supplemental Materials [file 41423_2022_887_MOESM1_ESM.docx]

**Supplementary Materials:**

**SARS-CoV-2 Virus NSP14 Impairs NRF2/HMOX1 Activation by Targeting Sirtuin 1**

Shilei Zhang, Jingfeng Wang, Lulan Wang, Saba Aliyari, Genhong Cheng^1^

Department of Microbiology, Immunology, and Molecular Genetics, University of California, Los Angeles, Los Angeles, CA 90095;

^1^To whom correspondence should be addressed: Dr. Genhong Cheng, Department of Microbiology, Immunology, and Molecular Genetics, University of California, Los Angeles, 615 Charles Young Drive, South Biomedical Science Research Building, Room 210A, Los Angeles, CA 90095. E-mail address: gcheng@mednet.ucla.edu

This file includes:

Figures S1 to S7

Legends for Figures S1 to S7


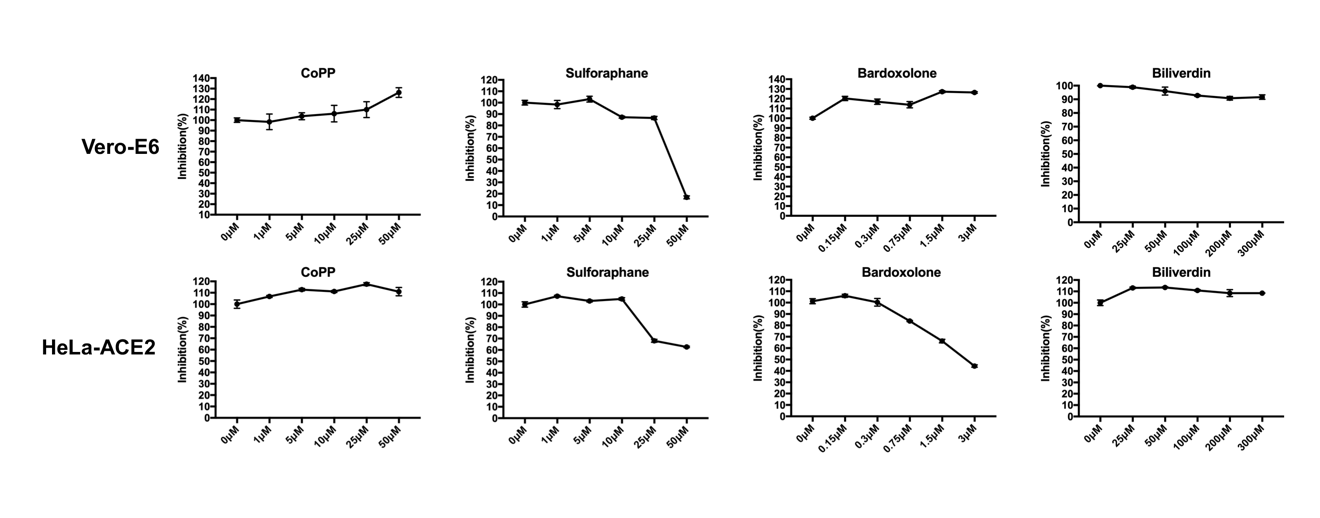


**FigS1. Effect of NRF2 agonists on cell viability.** The cell viability tests of Vero-E6 cells and HeLa-ACE2 cells 24 h after the NRF2 agonists treatment at different concentrations by using CCK8 kit.


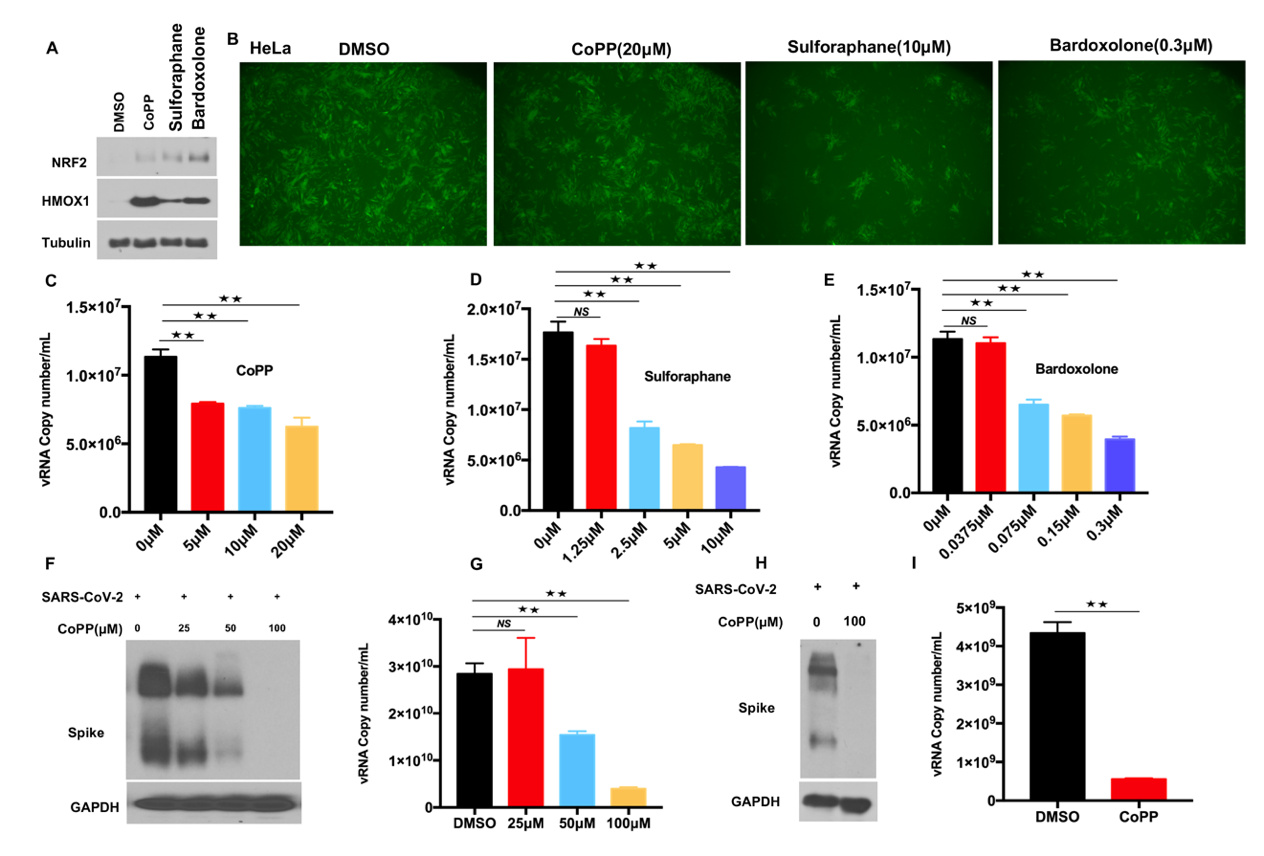


**FigS2. NRF2/HMOX1 agonists inhibit SARS-CoV-2 replication.** **(A)** NRF2/HMOX1 expression in Vero-E6 cells. Vero-E6 cells were stimulated with NRF2 agonists, CoPP(20μM), NRF2/Sulforaphane(10μM), and Bardoxolone(0.3μM) for 24 h. Immunoblotting analysis of NRF2 and HMOX1 were performed. **(B)** Microscopy images of GFP-SARS-CoV-2 replication in ACE2-HeLa cells. ACE2-HeLa cells infected with GFP-SARS-CoV-2 at 0.01 MOI were treated with NRF2 agonists at the indicated concentration for 24h. **(C-E)** ACE2-HeLa cells were infected with SARS-CoV-2 at 0.01 MOI and treated with increasing dose of NRF2 agonists for 24h. Viral copies in the cell supernatants were quantified by Real-time PCR analysis. Anti-SARS-CoV-2 activity of CoPP was also tested in Calu-3 cells **(F, G)** and Caco-2 cells **(H, I)**. SARS-CoV-2 infects Calu-3 and Caco-2 cells at the presence of CoPP with the indicated concentration for 24 h. Immunoblotting analysis of Spike and Real-time PCR quantification of viral copies in the supernatants were performed. Asterisks **(C, D, E, G, and I)** represent statistical significance based on two-tailed unpaired Student’s t test (*P < 0.05, **P < 0.01).


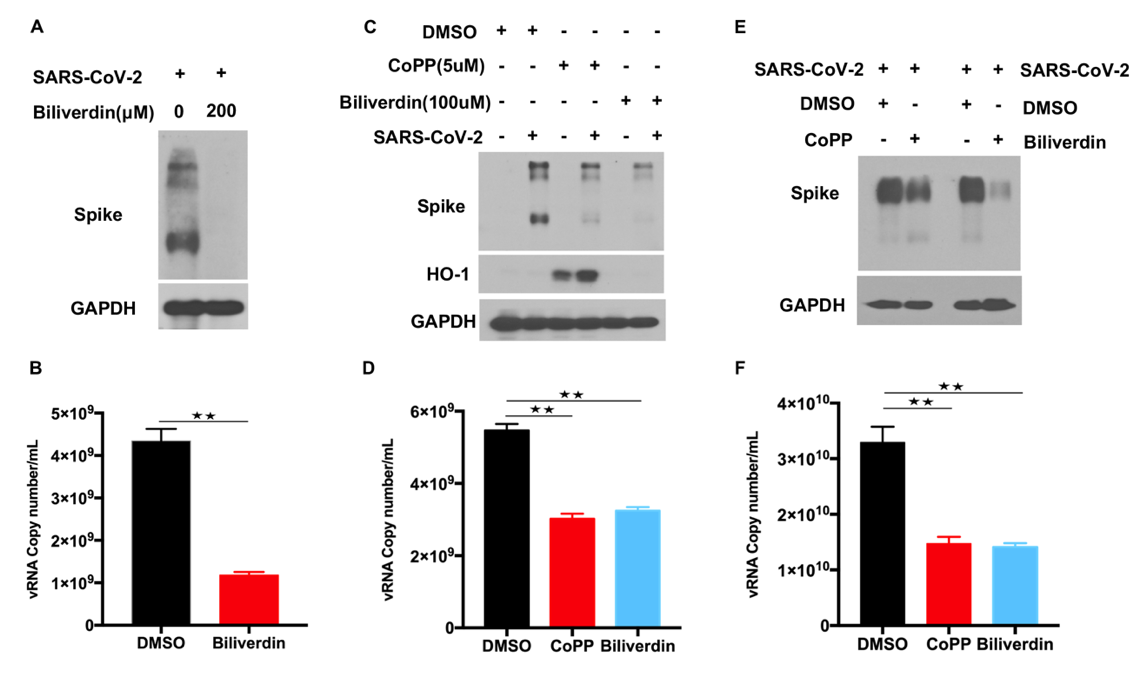


**FigS3. Biliverdin suppresses SARS-CoV-2 replication in different cell lines.** The anti-SARS-CoV-2 activity of Biliverdin were performed in different cell lines, including Caco-2**(A, B)**, Huh7**(C, D)**, and Vero-E6 cells **(E, F)**. These cell lines were infected with 0.01 MOI of SARS-CoV-2 for 24 h with or without biliverdin at the indicated dose. The expression of Spike was analyzed by western blot assay. The viral copies of N gene were measured by Real-time PCR. In immunoblotting analysis, GAPDH was used as loading control. Asterisks **(B, D, and F)** represent statistical significance based on two-tailed unpaired Student’s t test (*P < 0.05, **P < 0.01).


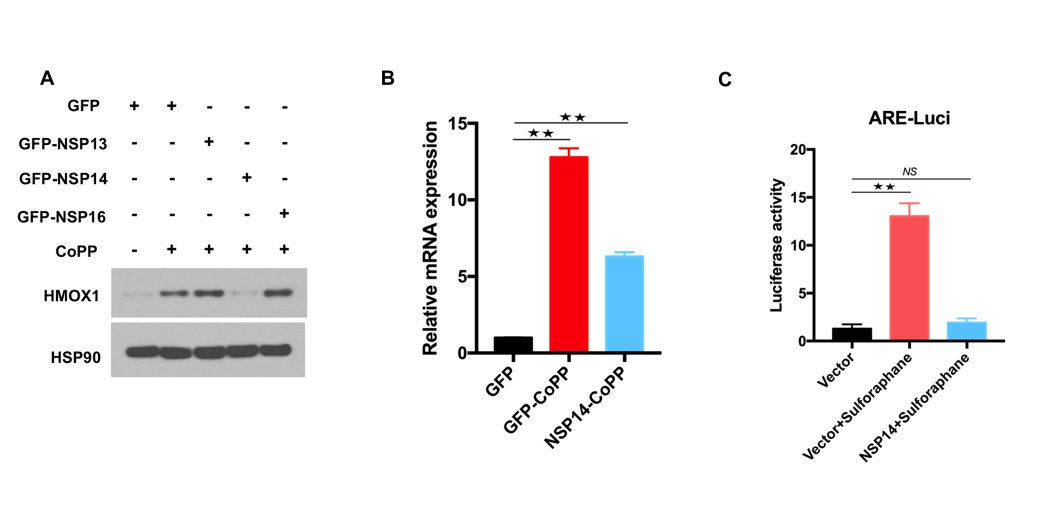


**FigS4. NSP14 inhibits HMOX1 expression. (A)** HEK293T cells were transfected with 400ng NSP13, NSP14, NSP16, and GFP vector control. At 24 h post-transfection, cells were subsequently stimulated with CoPP for additional 6 h. Cell lysates were harvested and subjected to immunoblotting analysis with the indicated antibodies. HSP90 was used as loading control. **(B)** HEK293T cells were introduced with 400ng NSP14 expressing plasmid or GFP control vector for 24 h, followed by additional 6 h stimulation with CoPP. mRNA expression of HMOX1 was analyzed by Real-time PCR assay. (C) HEK293T cells were transfected with Renilla luciferase control plasmid, firefly luciferase reporter plasmid ARE-Luc, and NSP14. At 24 h post transfection, cells were treated with Sulforaphane for additional 24 h, and then harvested for luciferase activity analysis. Asterisks **(B-C)** represent statistical significance based on two-tailed unpaired Student’s t test (*P < 0.05, **P < 0.01).


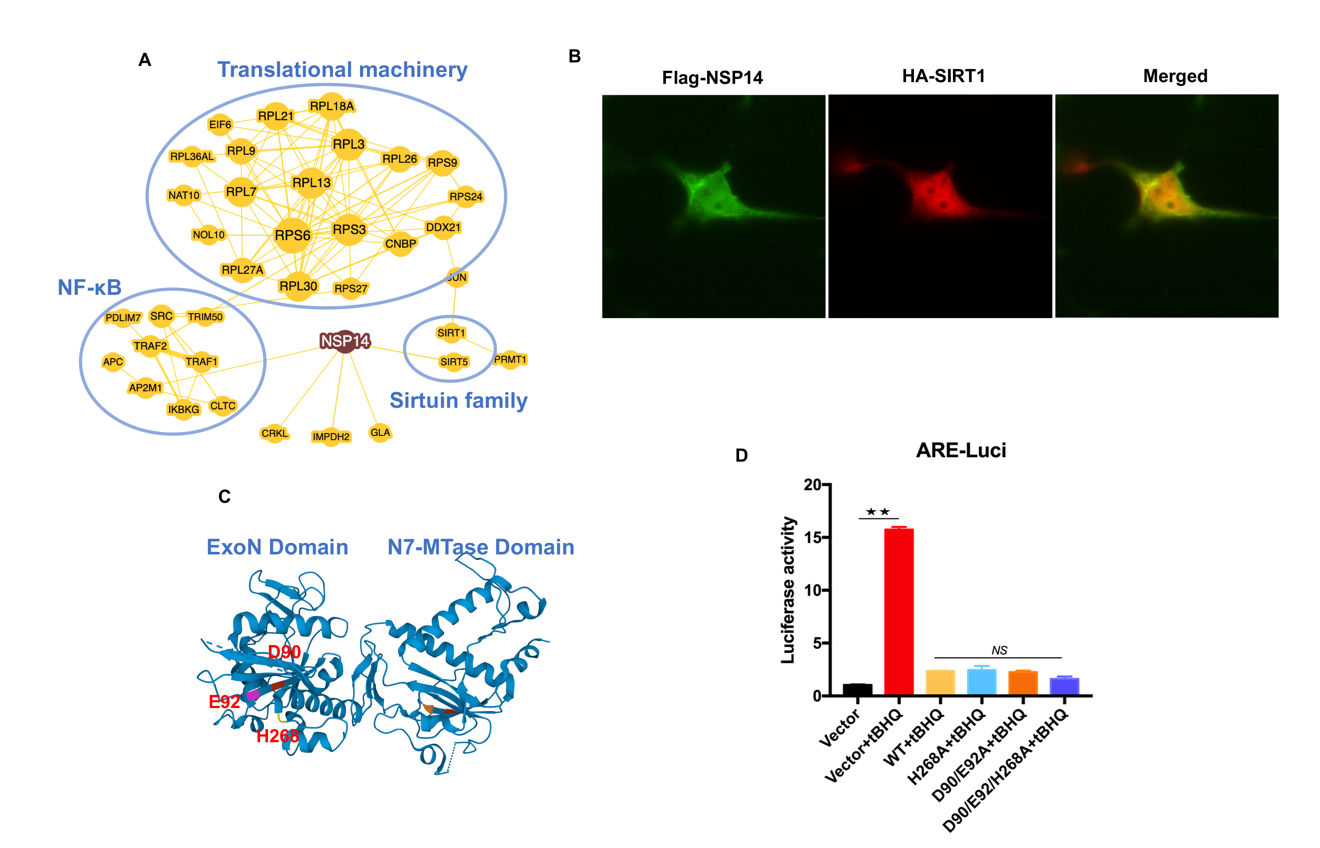


**FigS5. NSP14 interacts with SIRT1.** **(A)** Mapping of the protein-protein interaction network of NSP14 was performed by querying the protein-protein interaction database BioGRID. Each node represents a protein indicated by its protein symbol. Interactions of NSP14 and host proteins are indicated by yellow lines. Proteins in big blue circles mean that they were classified as a cluster by their function. **(B)** Immunofluorescence analysis of NSP14 and SIRT1 colocalization. HA-SIRT1 and Flag-NSP14 were co-transfected into HEK293T cells. After 24 h, cells were stained with specific antibody against Flag and HA, and subsequent with conjugated second antibodies. **(C)** Structure of NSP14 (DOI: 10.2210/pdb7QGI/pdb). The mutation sites used in (D) were highlighted with Red color. **(D-E)** Luciferase analysis of the inhibitory activities of NSP14 mutants on tBHQ-induced ARE activity.


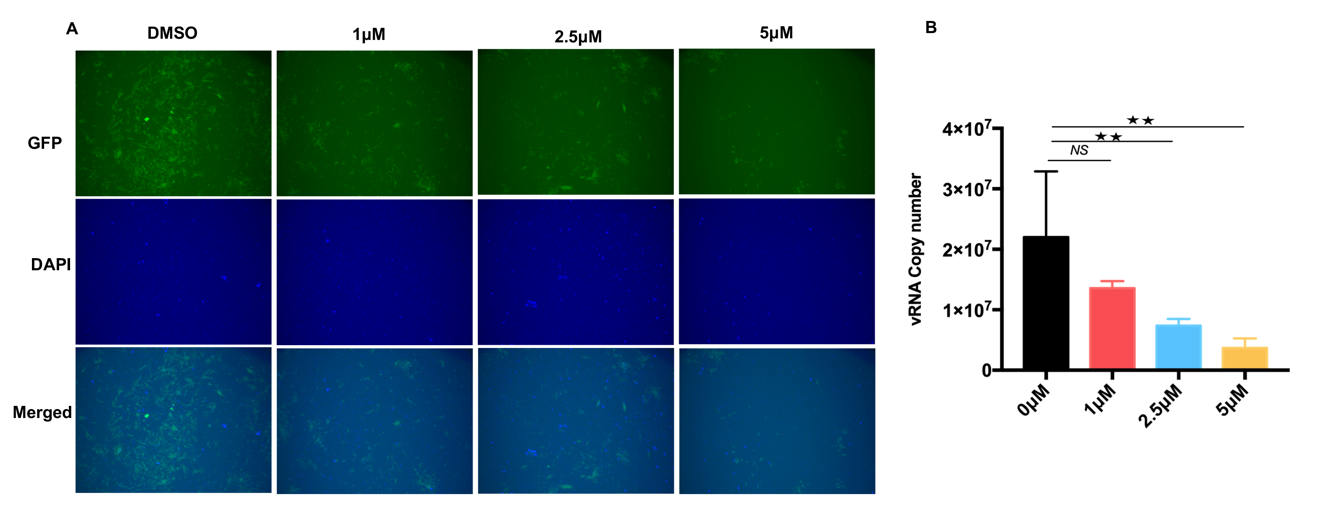


**FigS6. SIRT1 is a host restriction factor against SARS-CoV-2 replication.** ACE2-HeLa cells were infected with GFP-SARS-CoV-2 at 0.01 MOI in the treatment of increasing doses of SIRT1 agonist SRT1720 for 24 h. **(A)** Fluorescence microscopy images of virus replication were analyzed. The nuclei were stained with DAPI bye. **(B)** The viral yields in the cell supernatants were also quantified by Real-time PCR. Asterisks **(B)** represent statistical significance based on two-tailed unpaired Student’s t test (*P < 0.05, **P < 0.01).


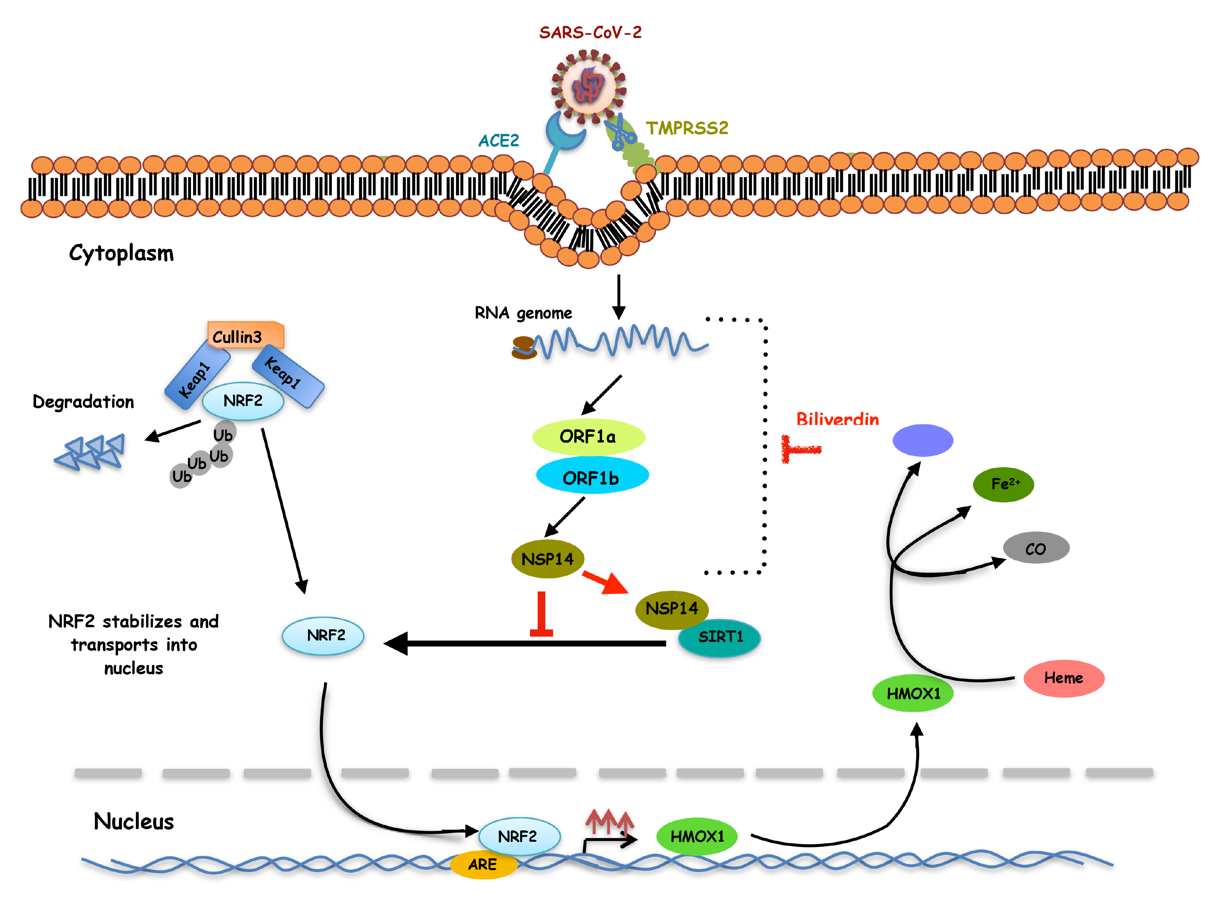


**FigS7. Proposed working model of NSP14 antagonizing the antiviral activity of NRF2/HMOX1.**
